# Canine Antibody Response to *Phlebotomus perniciosus* Bites Negatively Correlates with the Risk of *Leishmania infantum* Transmission

**DOI:** 10.1371/journal.pntd.0001344

**Published:** 2011-10-11

**Authors:** Michaela Vlkova, Iva Rohousova, Jan Drahota, Dorothee Stanneck, Eva Maria Kruedewagen, Norbert Mencke, Domenico Otranto, Petr Volf

**Affiliations:** 1 Department of Parasitology, Faculty of Science, Charles University in Prague, Prague, Czech Republic; 2 Bayer Animal Health GmbH, Leverkusen, Germany; 3 Department of Veterinary Public Health and Animal Sciences, Faculty of Veterinary Medicine of Bari, Bari, Italy; Lancaster University, United Kingdom

## Abstract

**Background:**

Phlebotomine sand flies are blood-sucking insects that can transmit *Leishmania* parasites. Hosts bitten by sand flies develop an immune response against sand fly salivary antigens. Specific anti-saliva IgG indicate the exposure to the vector and may also help to estimate the risk of *Leishmania* spp. transmission. In this study, we examined the canine antibody response against the saliva of *Phlebotomus perniciosus*, the main vector of *Leishmania infantum* in the Mediterranean Basin, and characterized salivary antigens of this sand fly species.

**Methodology/Principal Findings:**

Sera of dogs bitten by *P. perniciosus* under experimental conditions and dogs naturally exposed to sand flies in a *L. infantum* focus were tested by ELISA for the presence of anti-*P. perniciosus* antibodies. Antibody levels positively correlated with the number of blood-fed *P. perniciosus* females. In naturally exposed dogs the increase of specific IgG, IgG1 and IgG2 was observed during sand fly season. Importantly, *Leishmania*-positive dogs revealed significantly lower anti-*P. perniciosus* IgG2 compared to *Leishmania*-negative ones. Major *P. perniciosus* antigens were identified by western blot and mass spectrometry as yellow proteins, apyrases and antigen 5-related proteins.

**Conclusions:**

Results suggest that monitoring canine antibody response to sand fly saliva in endemic foci could estimate the risk of *L. infantum* transmission. It may also help to control canine leishmaniasis by evaluating the effectiveness of anti-vector campaigns. Data from the field study where dogs from the Italian focus of *L. infantum* were naturally exposed to *P. perniciosus* bites indicates that the levels of anti-*P. perniciosus* saliva IgG2 negatively correlate with the risk of *Leishmania* transmission. Thus, specific IgG2 response is suggested as a risk marker of *L. infantum* transmission for dogs.

## Author Summary

*Leishmania infantum* is the causative agent of zoonotic visceral leishmaniasis in the Mediterranean Basin and *Phlebotomus perniciosus* serve as the major vector. In the endemic foci, *Leishmania* parasites are transmitted mostly to dogs, the main reservoir host, and to humans. We studied the canine humoral immune response to *Phlebotomus perniciosus* saliva and its potential use as a marker of sand fly exposure and consequently as a risk marker for *Leishmania* transmission. We also characterized major salivary antigens of *P. perniciosus*. We demonstrated that under laboratory conditions, the levels of anti-*P. perniciosus* saliva antibodies positively correlated with the number of blood-fed sand flies and therefore, may be used to evaluate the need for, and the effectiveness of, anti-vector campaigns. In parallel, we studied sera of dogs naturally exposed to *P. perniciosus* in highly active focus of canine leishmaniasis in Southern Italy. Specific antibodies against *P. perniciosus* saliva were significantly increased according to the ongoing sand fly season. Moreover, the levels of anti-*P. perniciosus* antibodies in naturally bitten dogs negatively correlated with anti-*Leishmania* seropositivity. Thus, for dogs living in endemic areas, specific antibody response against saliva of the vector is an important marker for estimating the risk of *Leishmania* transmission.

## Introduction

*Leishmania infantum* (syn. *Leishmania chagasi*) is a protozoan parasite that causes zoonotic leishmaniasis, including the life-threatening visceral form, occurring also in the Mediterranean Basin. Parasites are transmitted by the bite of infected phlebotomine sand flies to dogs, the major host and the main domestic reservoir for human visceral leishmaniasis, or to humans. The clinical forms of canine leishmaniasis range from asymptomatic to lethal (reviewed in [1], [2]). Nonetheless, all seropositive infected dogs, including those without any clinical signs, can serve as a source of infection for sand flies in endemic areas [3], [4]. The major vector of canine leishmaniases in Mediterranean countries, including Italy, is *Phlebotomus perniciosus* [5], [6]. Control programs for human visceral leishmaniasis caused by *L. infantum* are primarily aimed at preventing sand flies from feeding on dogs to reduce *Leishmania* transmission among dogs and humans (reviewed in [1], [2]).

Measuring the exposure of dogs to sand fly bites is important for estimating the risk of *L. infantum* transmission. Recently, it was demonstrated that experimental exposure of dogs to *Lutzomyia longipalpis* bites elicits the production of specific anti-saliva IgG which positively correlates with the number of blood-fed sand flies [7]. Therefore, monitoring canine IgG levels specific for sand fly saliva could indicate the intensity of exposure to sand fly bites. Such a monitoring technique would be useful for evaluating the need for, and effectiveness of, anti-vector campaigns [7], [8].

Exposure to sand fly bites as well as immunization with sand fly saliva or its compounds elicits in naive hosts protection against *Leishmania* infection under laboratory conditions (reviewed in [9]). It is widely accepted that the protective effect is mediated by CD4^+^ Th1 cellular response and characterized by increased production of IFN- γ, which activates macrophages to kill *Leishmania* parasites (reviewed in [10]). Recently, it was shown that protective effect elicited by inoculation of *Lutzomyia longipalpis* recombinant proteins in dogs was associated with production of IFN-γ by CD3^+^ CD4^+^ T cells and by dominance of IgG2 antibodies [11].

In this study we described the anti-saliva IgG response in dogs experimentally exposed to *P. perniciosus* under laboratory conditions and those naturally exposed in an endemic focus of *L. infantum*. We also tested the association between the anti-saliva IgG subclasses and the levels of IFN-γ in *Leishmania infantum*-seropositive and -seronegative dogs. Additionally, we characterized the major *P. perniciosus* salivary antigens recognized by sera of experimentally and naturally bitten dogs.

## Methods

### Ethical statement

#### Experiments with dogs exposed to sand fly bites under laboratory conditions

Husbandry of animals in the Animal Center (Germany) complies with the European Commission guidelines for the accommodation of animals used for experimental and other scientific purposes - Commission Recommendation of 18 June 2007 (2007/526/EC). The compliance to aspects of animal welfare law is regularly monitored by the BAH animal welfare commissioner and the state veterinarian. The study design and the experimental procedures were approved by the responsible authorities (LANUV - Regional Authority for Nature, Environment and Consumer protection in North Rhine-Westphalia, Germany).

#### Experiments with dogs naturally exposed to sand fly bites

All procedures were approved by the Animal Ethics Committee from the Faculty of Veterinary Medicine, University of Bari, Italy and authorized by the Italian Ministry of Health (Authorization number 72/2009C n°69062; 28/11/08). Adverse events were individually registered in accordance to the International Cooperation on Harmonization of Technical Requirements for Registration of Veterinary Medicinal Products (VICH) and Good Clinical Practice (GCP) Guideline (GL9).

### Sand flies and salivary gland dissection

A colony of *Phlebotomus perniciosus* was reared under standard conditions as described in [12]. Salivary glands were dissected from 4–6 day old female sand flies, placed into 20 mM Tris buffer with 150 mM NaCl and stored at −20°C.

### Experimental exposure

Twelve laboratory dogs, beagles, were housed and handled in the Bayer Animal Health GmbH animal facility (Leverkusen, Germany). Dogs were sedated and individually exposed to approximately 200 *P. perniciosus* females as described in [7], [13]. Twenty hours after exposure, sand flies were collected and microscopically examined to assess the ratio of blood-fed females. In two independent experiments, two groups of three dogs each were used. Dogs in groups 2 and 4 wore insecticide-impregnated collars that were administrated 8 days before the first sand fly exposure, for a reduction of sand fly bites. In comparison, dogs in groups 1 and 3 remained without any repellent or insecticide application during the whole study. Therefore, dogs in groups 1 and 3 are hereafter defined as high-exposed (HE) and the dogs in groups 2 and 4 as low-exposed (LE). Dogs were exposed to sand fly bites once a week for five consecutive weeks. For the detailed numbers of blood-fed females see Table 1. Blood samples were collected throughout the study according to the following schedule: before the first exposure (week 0, pre-immune serum), during the sand fly sensitization (weeks 1–5), and weekly after the last exposure for 5 weeks (weeks 6–10).

**Table 1:** Numbers of blood-fed *Phlebotomus perniciosus* females per dog.

Week	Group 1	Group 2	Group 3	Group 4
1	221±5	49±15	173±8	27±4
2	191±47	125±69	155±18	11±6
3	188±7	61±20	125±6	36±15
4	156±4	39±11	169±12	20±3
5	195±9	83±36	158±11	8±1
average	190±10	71±16	156±6	20±4

(average ± standard error; groups 1, 3 – high-exposed dogs; groups 2, 4 – low-exposed dogs).

### Field study

Twenty nine mixed-breed young dogs (from 90 to 145 days old) and eleven laboratory reared beagles (120 days old) were enrolled in the trial. All animals were housed in a private open-air shelter in Putignano (Bari province, Apulia, Italy), where *P. perniciosus* is the most abundant phlebotomine sand fly species [14]. All dogs were vaccinated against common dog pathogens and dewormed as described in [15]. The canine antibody response against *P. perniciosus* saliva was studied at the beginning (March 2008) and at the end (November 2008) of the sand fly season. In parallel, at four intervals (March, July, November 2008 and March 2009) dogs were tested for *L. infantum* infection status by serological, cytological and molecular methods. All dogs were *L. infantum* negative at the beginning of the trial (March 2008), which was proved by all three diagnostic methods used. *Leishmania*-positive dogs were defined by positive anti-*L. infantum* serology and, in a subset of seropositive dogs (4 out of 18), the infection was confirmed by PCR or cytology. For details on the diagnostic methods, see [15], [16]. Considering the long incubation period of canine leishmaniasis and the occurrence of sand flies exclusively during the summer season (from June to October) [14], dogs with anti-*Leishmania* seroconversion in March (2009) are presumed to have become infected during the previous season (2008). Dogs that were seronegative for *L. infantum* at all four screening intervals were included in the *Leishmania*-negative group.

### Detection of anti – *P. perniciosus* saliva antibodies

Anti-*P. perniciosus* IgG, IgG1 and IgG2 were measured by enzyme-linked immunosorbent assay (ELISA) as described in [7] with some modification. Briefly, microtiter plates were incubated with 6% (w/v) low fat dry milk in PBS with 0.05% Tween 20 (PBS-Tw). Canine sera were diluted 1∶200 or 1∶500 in 2% (w/v) low fat dry milk/PBS-Tw. Secondary antibodies (anti-dog IgG, IgG1, or IgG2 from Bethyl laboratories) were diluted and incubated as previously described [7]. Absorbance was measured at 492 nm using a Tecan Infinite M200 microplate reader (Schoeller). The cut-off value (IgG = 0.145; IgG1 = 0.126; IgG2 = 0.165) was determined as less than two times the standard error of the mean of the absorbance of pre-immune serum.

### Western blot analysis

*Phlebotomus perniciosus* salivary gland homogenate from 5-day-old sand fly females were separated by SDS-PAGE on a 10% gel under non-reducing conditions using the Mini-Protean III apparatus (BioRad). Separated proteins were blotted onto a nitrocellulose (NC) membrane by Semi-Phor equipment (Hoefer Scientific Instruments) and blocked with 5% (w/v) low fat dry milk in Tris-buffered saline with 0.05% Tween 20 (TBS-Tw). Strips of NC membrane were incubated with canine sera diluted 1∶50 (experimentally bitten dogs) or 1∶25 (naturally bitten dogs) in TBS-Tw for 1 hour. The strips were then washed three times with TBS-Tw and incubated with peroxidase-conjugated sheep anti-dog IgG (Bethyl Laboratories) diluted 1∶3000 in TBS-Tw. The chromogenic reaction was developed using a solution containing diaminobenzidine and H_2_O_2_.

### Mass spectrometry

For mass spectrometric analysis, salivary glands from 5-day-old *P. perniciosus* females were homogenized by 3 freeze-thaw cycles. Samples were dissolved in non-reducing sample buffer and electrophoretically separated in 10% polyacrylamide SDS gel. Proteins within the gels were visualized by staining with Coomassie Blue G-250 (Bio-Rad). The individual bands were cut and incubated with 10 mM dithiothreitol (DTT) and then treated with 55 mM iodoacetamid. Washed and dried bands were digested with trypsin (5 ng Promega). The alpha-cyano-4-hydroxycinnamic acid was used as a matrix. Samples were measured using a 4800 Plus MALDI TOF/TOF analyzer (AB SCIEX). Peak list from the MS spectra was generated by 4000 Series Explorer V 3.5.3 (AB SCIEX) without smoothing. Peaks with local signal to noise ratio greater than 5 were picked and searched by local Mascot v. 2.1 (Matrix Science) against a database of putative salivary protein sequences derived from a cDNA library [17]. Database search criteria were as follows – enzyme: trypsin, taxonomy: *Phlebotomus*, fixed modification: carbamidomethylation, variable modification: methionine oxidation, peptide mass tolerance: 80 ppm, one missed cleavage allowed. Only hits that scored as significant (p<0.05) are included.

### Statistical analysis

The data from experimentally bitten dogs obtained by ELISA were subjected to GLM ANOVA and Scheffe's Multiple Comparison procedure to analyse differences in kinetics of antibody response between HE and LE dogs at all sampling points. The non-parametric Wilcoxon rank sum test for differences in medians was used for comparison of anti-*P. perniciosus* IgG, IgG1, IgG2 and IgG1/IgG2 ratios between *Leishmania*-seropositive and -seronegative dogs. The non-parametric Wilcoxon signed-rank test for differences in medians was used for comparison of antibody increases between March and November blood samples in naturally bitten dogs. For correlation tests we used the non-parametric Spearman rank correlation matrix. For all tests statistical significance was regarded as a p-value less than or equal to 0.05. All statistical analyses were performed using NCSS 6.0.21 software.

Relative risk (the probability of the developing the disease occurring in the group exposed to the risk factor versus a non-exposed group), attributive risk (absolute effect of exposure to the risk factor) and ODDS ratio (odds of an event occurring in the exposed group to the odds of it occurring in non-exposed group) were calculated for dogs from the field study to find out the relationship between the levels of anti-*P. perniciosus* saliva antibodies and leishmaniasis incidence as described in [18]. Low level of specific antibodies (lower than the cut-off value) was determined as the risk factor and the confidence interval for relative risk was calculated as described in [19].

### List of the protein accession numbers

*Phlebotomus perniciosus*: DQ153102; DQ154099; DQ150622; DQ150621; DQ192490; DQ192491; DQ153100; DQ153101; DQ153104; DQ150624; DQ150623; DQ150620; DQ153105.

*Lutzomyia longipalpis*: AF132518.

## Results

### Antibody response in experimentally bitten dogs

To investigate the kinetics of antibody response against anti-*P. perniciosus* saliva, two groups of experimentally bitten dogs, low-exposed (LE) and high-exposed (HE), were followed for 10 weeks. Five weekly experimental exposures to *P. perniciosus* bites led to increased levels of anti-saliva specific IgG, IgG1 and IgG2 in both LE and HE groups. No anti-saliva antibodies were detected in any pre-immune dog sera tested.

**Figure 1:**
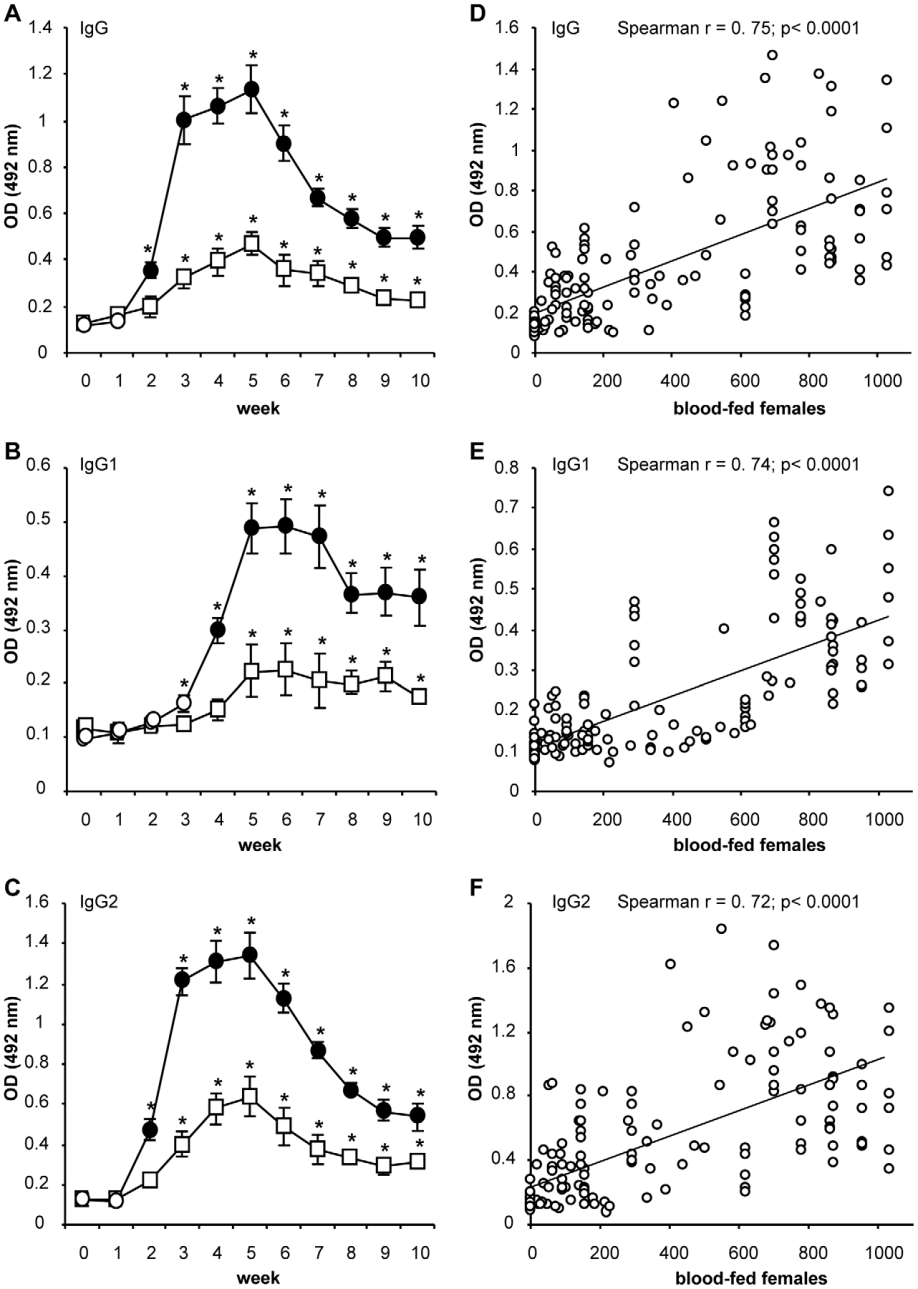
Anti-sand fly saliva antibody response in dogs experimentally bitten by *Phlebotomus perniciosus*. (A–C) Beagle dogs (3 per group) were divided into low-exposed (square) and high-exposed groups (circles) and were exposed to sand fly bites in weeks 1–5. For detailed numbers of blood-fed females see Table 1. Levels of specific IgG (A); IgG1 (B); and IgG2 (C) were measured by ELISA (at 492 nm) in all canine pre-immune and immune sera. Full circles represent significant difference between high- and low-exposed dogs (p<0.05); asterisks indicate significant difference (p<0.05) compared to pre-immune sera. Data are presented as the means ± standard errors of the means from two independent studies. (D–F) Correlation between number of blood-fed sand fly females and the levels of canine anti-*P. perniciosus* IgG (D); IgG1 (E); and IgG2 (F) was performed using Spearman Rank Correlation Matrix. OD = optical density.

In HE dogs, anti-*P. perniciosus* antibody levels increased significantly (p<0.05) in comparison to the pre-immune sera after the second (IgG; IgG2) and third exposure (IgG1) (Figure 1A–C). Anti-saliva IgG and IgG2 developed with similar kinetics; rapidly increased after the third exposure, and gradual increase until week five (the last exposure), followed by a steady decrease to the end of the study. Anti-saliva IgG1 increased rapidly between weeks three and five and persisted at elevated levels until the end of the study.

In LE dogs, anti-*P. perniciosus* antibody levels increased significantly (p<0.05) in comparison to the pre-immune sera after the fourth (IgG; IgG2) and sixth exposure (IgG1) (Figure 1A–C). Similar to HE dogs, kinetics of anti-*P. perniciosus* IgG and IgG2 in LE dogs was detected at peak levels on week five followed by a rapid decrease. Conversely, IgG1 was measured at peak levels on week six and persisted at elevated quantities to the end of the study (Figure 1A–C).

All HE dogs produced significantly higher levels of anti-*P. perniciosus* IgG (p = 0.0001), IgG1 (p = 0.0032) and IgG2 (p = 0.0003) compared to LE dogs throughout the study (Figure 1A–C). A positive correlation was detected between number of blood-fed female sand flies and the levels of canine anti-*P. perniciosus* IgG (r = 0.75, p<0.0001), IgG1 (r = 0.74, p<0.0001) and IgG2 (r = 0.72, p<0.0001) (Figure 1D–F). Overall, sera of experimentally bitten dogs produced higher concentrations of specific IgG2 compared to specific IgG1 (data not shown).

### Antibody response in naturally bitten dogs

**Figure 2:**
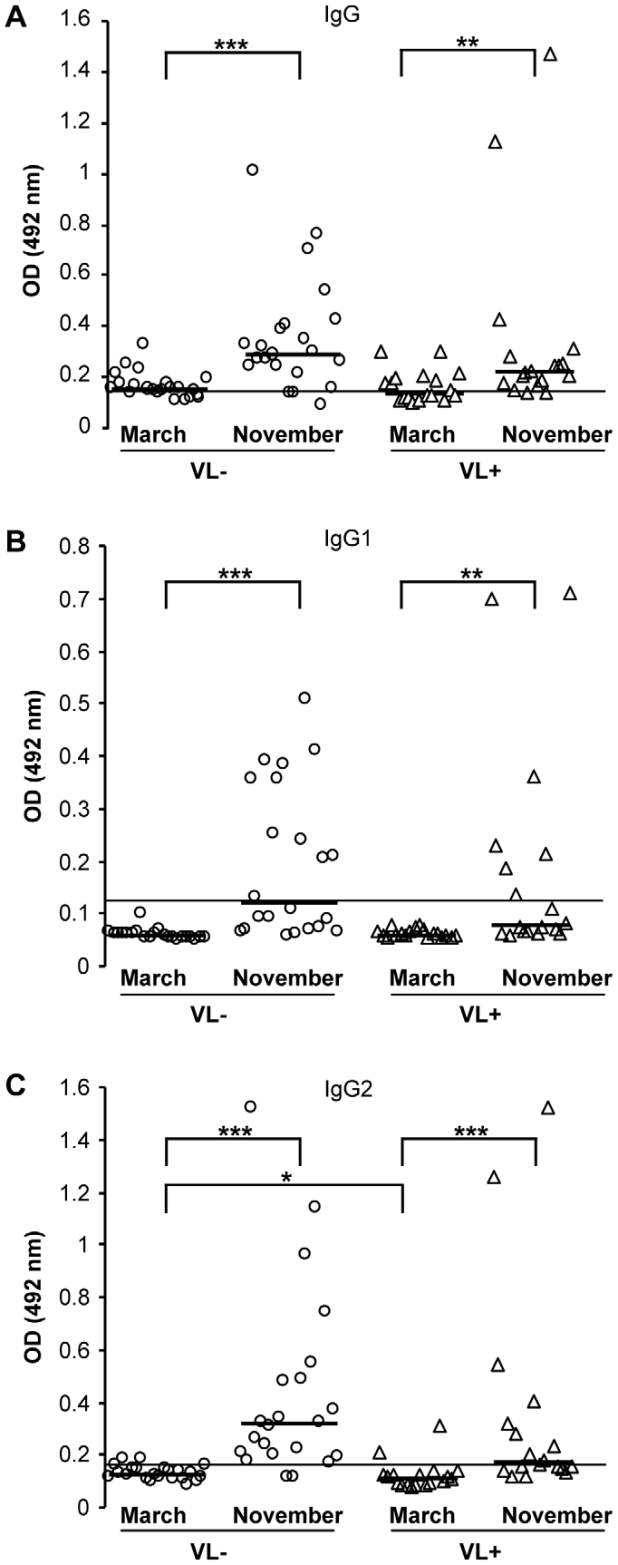
Anti-sand fly saliva antibody response in dogs naturally bitten by *Phlebotomus perniciosus*. Anti-*P. perniciosus* IgG (A); IgG1 (B) and IgG2 (C) response was measured in sera of naturally bitten dogs from endemic area of visceral leishmaniasis. All dogs were *Leishmania infantum* seronegative at the beginning of the trial. ELISA was performed against *P. perniciosus* salivary gland homogenate using canine sera from *Leishmania infantum*-seropositive dogs (open triangle, n = 18) and *Leishmania*-seronegative dogs (open circles, n = 22). Serum samples were taken at the beginning (March) and at the end of the sand fly season (November). The symbols indicate results of each serum tested, bars represent median values of the groups. Lines represent cut-off values (two times the standard error of the mean of the absorbance of experimentally bitten dog pre-immune sera). Asterisks indicate statistical significance between *Leishmania*-seropositive and -seronegative dogs and significant increase of antibodies during the sand fly season within the group (* p<0.05; ** p<0.01; *** p<0.001). OD = optical density.

To determine the anti-*P. perniciosus* saliva antibody levels and the seasonal changes in specific antibody response, canine sera were screened at the beginning and at the end of the sand fly season, March and November, respectively. Incidence of leishmaniasis in dogs naturally exposed to sand flies was high, 18 out of 40 (45%) were found anti-*L. infantum* seropositive (0/40 in March 2008; 0/40 in July 2008; 5/40 in November 2008; 13/40 in March 2009). In March, higher levels of anti-*P. perniciosus* IgG and IgG2 (compared to cut-off value) were detected in about 55% and 10% of dog sera, respectively, while IgG1 levels were comparable to pre-immune sera (Table 2). In November, elevated levels of specific IgG were found in 87.5%, IgG2 in 72.5% and IgG1 in 45% of the 40 enrolled dogs (Table 2). In both groups of dogs, *Leishmania*-positive and *Leishmania*-negative, specific IgG, IgG1 and IgG2 levels significantly increased during the sand fly season (Figure 2A–C).

**Table 2:** Numbers of dogs positive for anti-*Phlebotomus perniciosus* antibodies in *Leishmania infantum*-seropositive and -seronegative dogs.

	Leishmania negative dogs (n = 22)			Leishmania positive dogs (n = 18)		
	March	November	Increase(%)	March	November	Increase (%)
IgG	14	19	144^***^	8	15	104^**^
IgG1	0	11	235^***^	0	7	220^**^
IgG2	2	20	249^***^	2	9	205^***^
IgG1/IgG2^a^	0.47^*^	0.54	15	0.57^*^	0.73	28

(^a^ – significant difference in IgG1/IgG2 ratio between *Leishmania*-seropositive and -seronegative groups; *** p<0.001; ** p<0.01; * p<0.05).

*Leishmania*-positive and *Leishmania*-negative dogs did not statistically differ in IgG and IgG1 production (Figure 2A, B); however, a significant difference was found in IgG2 levels (Figure 2C). Indeed, *Leishmania*-positive dogs revealed significantly lower anti-*P. perniciosus* IgG2 at the beginning (p = 0.047) and at the end (p = 0.05) of sand fly season (Figure 2C). Negative correlation was found between the levels of anti-*P. perniciosus* saliva IgG2 and the risk of *Leishmania* transmission, supported well by epidemiological parameters: relative risk = 2.6 (95% confidence interval: 0.66; 10.63); attributive risk = 1.6; and ODDS ratio = 10. Sera of all naturally bitten dogs showed significantly higher levels of specific IgG2 compared to specific IgG1 (data not shown). Moreover, the IgG1/IgG2 ratio differed between *Leishmania*-positive and -negative dogs; *Leishmania*-positive dogs revealed higher IgG1/IgG2 ratio, although the difference was statistically significant only at the beginning of sand fly season (p = 0.039) (Table 2). Furthermore, higher levels of IFN-γ were detected in sera of *Leishmania*-negative dogs throughout the study but with no statistically significant difference (Figure S1).

### Identification and characterization of *P. perniciosus* salivary antigens

**Figure 3:**
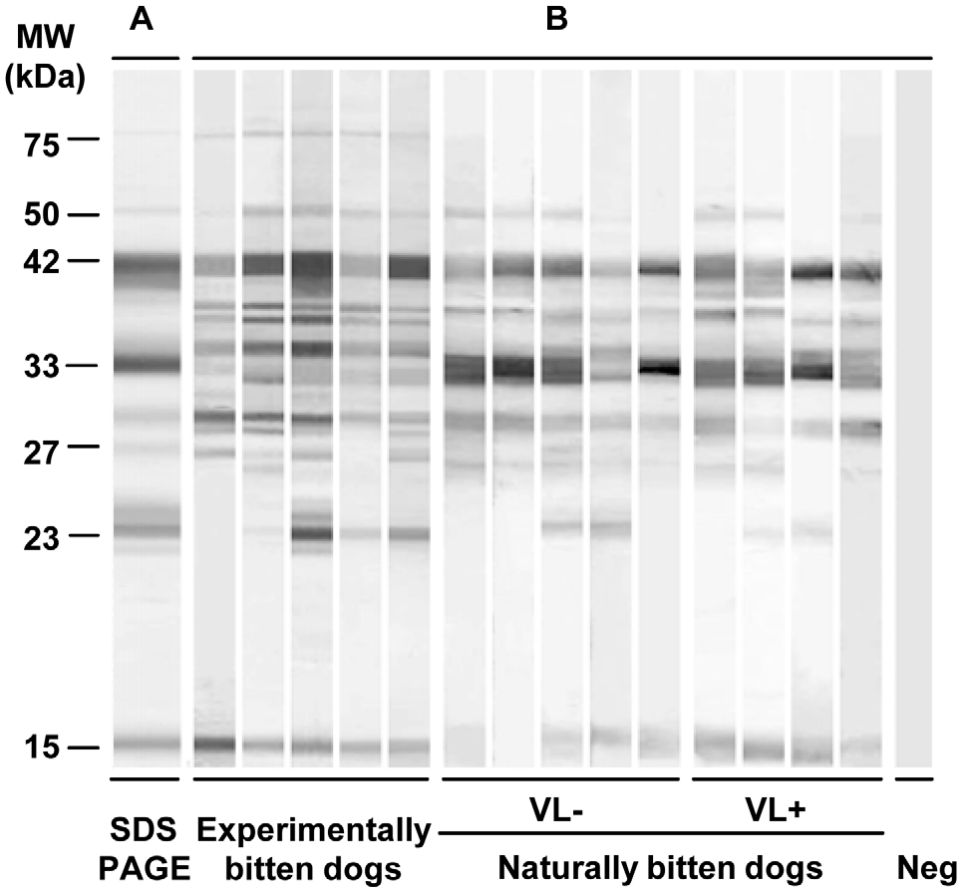
Anti-sand fly saliva antibody response in dogs experimentally and naturally bitten by *Phlebotomus perniciosus*. (A) Total protein profile, Commassie blue-stained SDS-PAGE gel after electrophoresis of *P. perniciosus* salivary gland homogenate. (B) Western blot of *P. perniciosus* salivary proteins recognized by sera of repeatedly bitten dogs. Western blot analysis was performed by sera of experimentally and naturally bitten dogs: *Leishmania infantum*-seronegative (VL−) and *L. infantum*-seropositive (VL+). Pre-immune serum of experimentally bitten dog was used as negative control (Neg).

*Phlebotomus perniciosus* salivary antigens were studied using sera of naturally and experimentally bitten dogs. Pre-immune sera of experimentally bitten dogs did not recognize any of the salivary proteins by Western blot analysis (Figure 3).

Sera of experimentally exposed dogs produced 11 bands on a salivary gland Western blot with approximate molecular weights of 75, 50, 42, 40, 38, 34, 33, 29, 27, 23 and 14 kDa (Figure 3). The molecular weights of salivary antigens recognized by canine sera were similar in all dogs tested with the exception of the 23 and 27 kDa protein bands (recognized only by some sera). The salivary gland antigens most intensely recognized by the sera of all experimentally bitten dogs had molecular weights of 42, 38, 33 and 29 kDa.

Sera of naturally bitten dogs with both negative and positive anti-*L. infantum* serology reacted with up to 9 protein bands of 50, 42, 38, 34, 33, 29, 27, 23 and 14 kDa. All naturally exposed dogs tested in both groups recognized similar salivary antigens and the most intensive reactions were detected with the 42 and 33 kDa salivary antigens.

Mass spectrometry revealed that the main antigens recognized by sera of bitten dogs were salivary endonuclease (50 kDa - DQ154099), yellow proteins (42 kDa - DQ150622; 40 kDa - DQ150621), apyrases (38 kDa - DQ192490; 38 kDa - DQ192491; 33 kDa - DQ192491), antigen-5 protein (29 kDa - DQ153101), D7 proteins (27 kDa - DQ153104; 23 kDa - DQ150624; 23 kDa - DQ150623, and proteins of the SP-15 like protein family (14 kDa - DQ150620; 14 kDa - DQ153105) (Table 3).

**Table 3:** *Phlebotomus perniciosus* salivary proteins recognized by sera of bitten dogs.

MW (kDa)	NCBI acc. number	Best match to NR protein database		
		Sequence name	E-value	Comments
75	DQ153102	29 kDa salivary protein (PpeSP08)	2.2e-6	unknown
50	DQ154099	41 kDa salivary protein (PpeSP32)	3.5e-9	endonuclease
42	DQ150622	43 kDa yellow-related salivary protein (PpeSP03B)	1.1e-68	yellow protein
40	DQ150621	42 kDa yellow-related salivary protein (PpeSP03)	4.5e-54	yellow protein
38	DQ192490	35.5 kDa salivary protein (PpeSP01)	5.6e-54	apyrase
38	DQ192491	35.3 kDa salivary protein (PpeSP01B)	0.035	apyrase
34	DQ153100	33 kDa salivary protein (PpeSP06)	2.2e-24	unknown
33	DQ192491	35.3 kDa salivary protein (PpeSP01B)	2.8e-72	apyrase
33	DQ153102	29 kDa salivary protein (PpeSP08)	0.0019	unknown
29	DQ153101	30 kDa antigen 5-related salivary protein (PpeSP07)	1.4e-12	Ag 5 protein
27	DQ153104	27 kDa D7-related salivary protein (PpeSP10)	0.0012	D7 protein
23	DQ150624	27 kDa D7-related salivary protein (PpeSP04B)	1.8e-16	D7 protein
23	DQ150623	24.5 kDa D7-related salivary protein (PpeSP04)	0.0069	D7 protein
14	DQ150620	14.8 kDa salivary protein (PpeSP02)	2.2e-13	SP15 like protein
14	DQ153105	13 kDa salivary protein (PpeSP11)	4.5e-15	SP15 like protein

## Discussion

Canine antibody response against *P. perniciosus* saliva was studied in dogs bitten by sand flies under well-defined laboratory conditions as well as in dogs from an endemic focus of visceral leishmaniasis in Italy.

In experimentally bitten dogs we observed a significant increase in production of specific IgG, IgG1 and IgG2 in the course of 10 weeks and a positive correlation was found between the levels of specific antibodies and the number of blood-fed females *P. perniciosus*. Anti-saliva specific IgG and IgG2 developed with similar kinetics and correspond well with previous results [7] in dogs experimentally bitten by *Lutzomyia longipalpis*. While in sera of healthy dogs, IgG1 and IgG2 usually occur in comparable concentrations [20], IgG2 prevailed in sera of bitten dogs in our study as well as in dogs experimentally bitten by *L. longipalpis* [7], [11].

In our field trial, we detected the increase in number of anti-*P. perniciosus* saliva seropositive dogs as well as in the amount of specific antibodies in dog sera as the sand fly season progressed. Statistically significant increases in production of specific IgG, IgG1 and IgG2 were observed in both *Leishmania*-positive and *Leishmania*-negative dogs at the end of sand fly season. Interestingly, *Leishmania*-positive dogs revealed significantly lower anti-*P. perniciosus* saliva IgG2 compared to *Leishmania*-negative dogs and the IgG1/IgG2 ratio was significantly higher in *Leishmania*-positive dogs. These data may suggest either that dogs with low IgG2 levels were at the higher risk of becoming *Leishmania*-infected or that *Leishmania* infection decreases the production of IgG2 in bitten dogs. Considering the IFN-γ levels in canine sera, that were shown to positively correlate with the protective Th1 immune response [11], it seems that the first hypothesis is more feasible. Although, the difference in IFN- γ production between *Leishmania*-negative and *Leishmania*–positive dogs was not statistically significant.

Published data from field studies suggests that humoral immune responses against sand fly saliva vary between hosts with cutaneous and visceral forms of leishmaniases (reviewed in [9], [21]). In foci of cutaneous leishmaniases caused by *L. tropica* and *L. braziliensis*, the levels of specific anti-sand fly saliva antibodies in humans positively correlated with the risk of *Leishmania* transmission [22], [23]. In contrast, in foci of visceral leishmaniasis caused by *L. infantum*, levels of human anti-sand fly saliva antibodies positively correlated with anti-*Leishmania* DTH (delayed-type hypersensitivity) and thus with protection against potential infection [24], [25]. So far, those studies have been performed only in humans. In canids, several studies showed presence of anti-sand fly saliva antibodies in sera from endemic areas in Brazil [8], [26], [27], however our study is the first describing the association with canine leishmaniasis.

Canine sera recognized more than eleven *P. perniciosus* antigenic bands by Western blot and the most intense reaction was often observed against a 42 kDa band. Mass spectrometry identified the 42 kDa band as a single protein belonging to the Yellow protein family (DQ150622). Previously, another Yellow protein of 47.3 kDa (AF132518) was reported as the major antigen recognized by sera of dogs bitten by *L. longipalpis* in the field [26]. The recombinant *L. longipalpis* Yellow proteins (rLJM11 and rLJM17) prepared in mammalian expression system kept their antigenicity and were successfully used to screen dog sera from Brazil [27], predicting similar features for Yellow protein of *P. perniciosus*. All canine sera tested recognized additional three major antigens of the 38, 33 and 29 kDa; the 38 and 33 kDa proteins are apyrases and the 29 kDa antigen represents the antigen 5-related protein family. These four antigens (42, 38, 33 and 29 kDa) are promising candidates as markers of sand fly exposure.

In conclusion, we confirmed that levels of antibodies against sand fly saliva positively correlate with the number of blood-fed sand flies and therefore, monitoring canine antibody response to specific sand fly salivary proteins may evaluate the need for, and effectiveness of, anti-vector campaigns. Moreover, this is the first study demonstrating relationship between the anti-sand fly saliva antibodies and the status of *L. infantum* infection in dogs. The levels of anti-*P. perniciosus* IgG2 in dogs naturally bitten by this sand fly species negatively correlate with the anti-*Leishmania* seropositivity. Thus, for dogs living in endemic area specific IgG2 response against saliva of the vector is suggested as a risk marker of *L. infantum* transmission.

## Supporting Information

Figure S1 **IFN-γ in the sera of** ***Leishmania infantum*** **-seropositive and -seronegative dogs naturally bitten by** ***Phlebotomus perniciosus*** **during the sand fly season.** Concentrations of IFN-γ were measured by ELISA using the Quantikine canine IFN-γ immunoassay (R&D Systems) following the manufacturer's guidelines. Serum samples, standards and controls were added without any dilutions. Absorbance was measured at 450 nm using a Tecan Infinite M200 microplate reader (Schoeller). Data were transformed and assessed as described in manufacturer's instructions (R&D Systems).
(TIF)
