# Supplementary figures and images for: Canine Antibody Response to Phlebotomus perniciosus Bites Negatively Correlates with the Risk of Leishmania infantum Transmission
Source: PLoS Negl Trop Dis. 2011 Oct 11;5(10):e1344. doi: 10.1371/journal.pntd.0001344 (PMC3191129; doi:10.1371/journal.pntd.0001344)

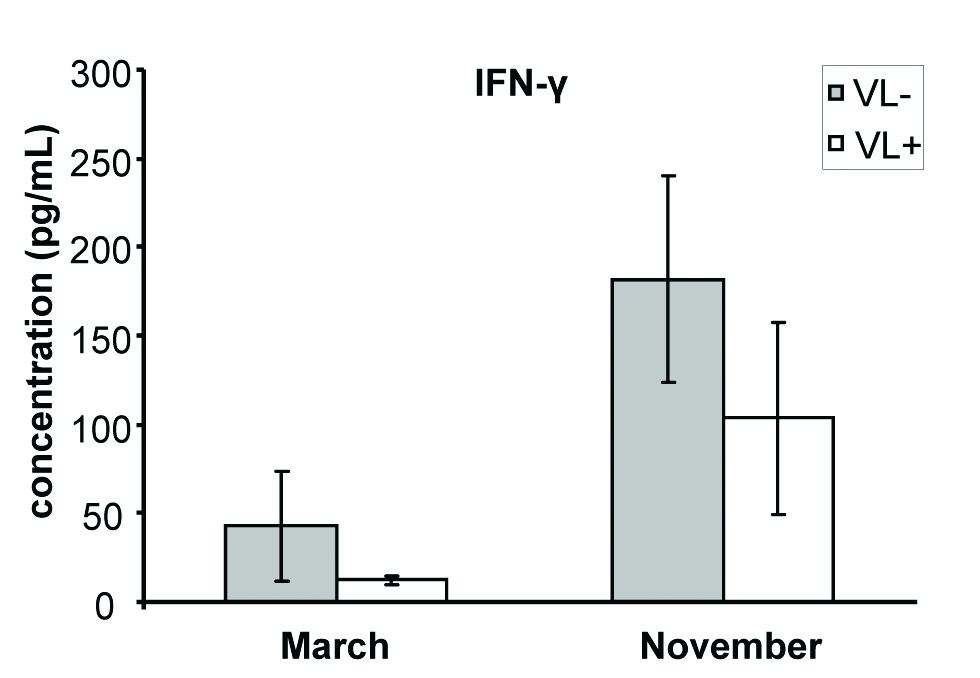

Supplement: Figure S1 — IFN-γ in the sera of Leishmania infantum -seropositive and -seronegative dogs naturally bitten by Phlebotomus perniciosus during the sand fly season. Concentrations of IFN-γ were measured by ELISA using the Quantikine canine IFN-γ immunoassay (R&D Systems) following the manufacturer's guidelines. Serum samples, standards and controls were added without any dilutions. Absorbance was measured at 450 nm using a Tecan Infinite M200 microplate reader (Schoeller). Data were transformed and assessed as described in manufacturer's instructions (R&D Systems). (TIF) [file pntd.0001344.s001.tif]
